# Development of multi-specific humanized llama antibodies blocking SARS-CoV-2/ACE2 interaction with high affinity and avidity

**DOI:** 10.1080/22221751.2020.1768806

**Published:** 2020-05-22

**Authors:** Jianbo Dong, Betty Huang, Zhejun Jia, Bo Wang, Sachith Gallolu Kankanamalage, Allison Titong, Yue Liu

**Affiliations:** a Ab Studio Inc., Hayward, CA, USA; b Ab Therapeutics Inc., Hayward, CA, USA

**Keywords:** SARS-CoV-2, COVID-19, bi-specific antibody, tri-specific antibody, llama antibody, humanized antibody, nanobody

## Abstract

Coronaviruses cause severe human viral diseases including SARS, MERS and COVID-19. Most recently SARS-CoV-2 virus (causing COVID-19) has led to a pandemic with no successful therapeutics. The SARS-CoV-2 infection relies on trimeric spike (S) proteins to facilitate virus entry into host cells by binding to ACE2 receptor on host cell membranes. Therefore, blocking this interaction with antibodies are promising agents against SARS-CoV-2. Here we describe using humanized llama antibody VHHs against SARS-CoV-2 that would overcome the limitations associated with polyclonal and monoclonal combination therapies. From two llama VHH libraries, unique humanized VHHs that bind to S protein and block the S/ACE2 interaction were identified. Furthermore, pairwise combination of VHHs showed synergistic blocking. Multi-specific antibodies with enhanced affinity and avidity, and improved S/ACE2 blocking are currently being developed using an *in-silico* approach that also fuses VHHs to Fc domains. Importantly, our current bi-specific antibody shows potent S/ACE2 blocking (KD – 0.25 nM, IC100 ∼ 36.7 nM, IC95 ∼ 12.2 nM, IC50 ∼ 1 nM) which is significantly better than individual monoclonal VHH-Fcs. Overall, this design would equip the VHH-Fcs multiple mechanisms of actions against SARS-CoV-2. Thus, we aim to contribute to the battle against COVID-19 by developing therapeutic antibodies as well as diagnostics.

Coronaviruses are enveloped, positive-sense single-strand RNA viruses with mammalian and avian hosts. Previous coronaviruses are known to infect humans include 229E, NL63, OC43, HKU1, SARS-CoV, and MERS-CoV, which cause a range of mild seasonal illnesses to severe diseases outbreaks. Notably, the past outbreaks of severe acute respiratory syndrome (SARS) (2003) and the Middle East respiratory syndrome (MERS) (2012) were caused by the coronaviruses SARS-CoV and MERS-CoV, respectively [1]. SARS-CoV-2, that emerged in December 2019, is the seventh known coronavirus to infect humans, and the third coronavirus to cross species barriers and cause severe respiratory infections in humans in less than two decades after SARS and MERS. It causes the coronavirus disease 2019 (COVID-19) [2] that is more contagious than SARS-CoV and MERS-CoV.

The high rate of infection and worldwide impact caused by the disease led the World Health Organization to declare COVID-19 a pandemic. As of 13th April 2020, the virus had confirmed infections in more than 1.8 million people worldwide, with nearly 120,000 confirmed deaths and an estimated 6.34% mortality rate [3]. Identification of the aetiology of the virus, publication of studies, and international collaborative efforts have led to the rapid development of real-time PCR diagnostic assays that support case ascertainment and tracking of COVID-19 outbreak. However, validated serologic diagnostic assays and therapeutics against SARS-CoV-2 are still lacking, and have become an urgent necessity to combat COVID-19.

The SARS-CoV-2 virion consists of a helical capsid formed by nucleocapsid (N) proteins bound to the RNA genome, that is enclosed by membrane (M) proteins, envelope (E) proteins and trimeric spike (S) proteins that render them their “corona-like” appearance [4]. The S protein receptor-binding domain (RBD) binds to the angiotensin-converting enzyme (ACE2) on the cell membranes of type 2 pneumocytes and intestinal epithelial cells. Following binding, the S protein is cleaved by host cell transmembrane serine protease 2 (TMPRSS2), that facilitates subsequent viral entry into the host cell [5].

Therapeutic antibodies are known to protect against viral infections via two mechanisms of action, i.e. Fc-independent functions that block virus/host receptor interaction, and induce virus aggregation, and Fc-dependent functions that cause Fc-FcR interaction to activate immune cells leading to the killing of the virus [6]. In general, combinations of antibodies targeting multiple epitopes have better viral neutralizing ability than single monoclonal antibodies [7]. However, immunoglobulin isolation from COVID-19 survivors is limited by the lack of availability of plasma from donors, and combinatorial treatment with several monoclonal antibodies is also limited due to high cost of production and potential toxicity. In order to overcome these existing limitations, we employed a novel approach of using humanized llama antibodies that blocks the interaction of SARS-CoV-2 S protein and ACE2, with the goal of rapidly developing high affinity and avidity bi- or tri-specific therapeutic antibodies that neutralize SARS-CoV-2 before it infects cells. In addition, previous reports have shown that if viruses are bound by low titre therapeutic antibodies with low affinity and avidity, the Fc-FcR interaction might trigger antibody-dependent enhancement (ADE) of virus entry into host cells [8]. Therefore, we also aimed to circumvent ADE by developing high titre neutralizing antibodies.

We used one naïve and one designed synthetic llama VHH library for this approach. The naïve library was constructed with PBMCs from 65 llamas, and the synthetic library was constructed from the naïve VHH library, where the VHH framework was partially humanized and the CDR1, 2, and 3 of VHH were shuffled to generate enhanced diversity and keep low immunogenicity. We panned the two llama VHH libraries against recombinant SARS-CoV-2 S protein. After panning, we obtained 91 high-affinity VHH hits for SARS-CoV-2 S protein binding, among which, 69 were unique sequences. We also assessed the ability of VHHs to block the S/ACE2 interaction *in-vitro* and discovered that 15 out of 69 unique S protein binders had S/ACE2 blocking function, top 9 of them are listed in Figure 1. Follow-up studies revealed that pairwise combination of some of the 9 VHH blockers led to synergistic blocking efficacy of S/ACE2 interaction. Notably, a combination of VHH1 with any other VHH did not affect its blocking function, making it the possible base unit in multi-specific antibody design (Figure 1).

**Figure 1.**
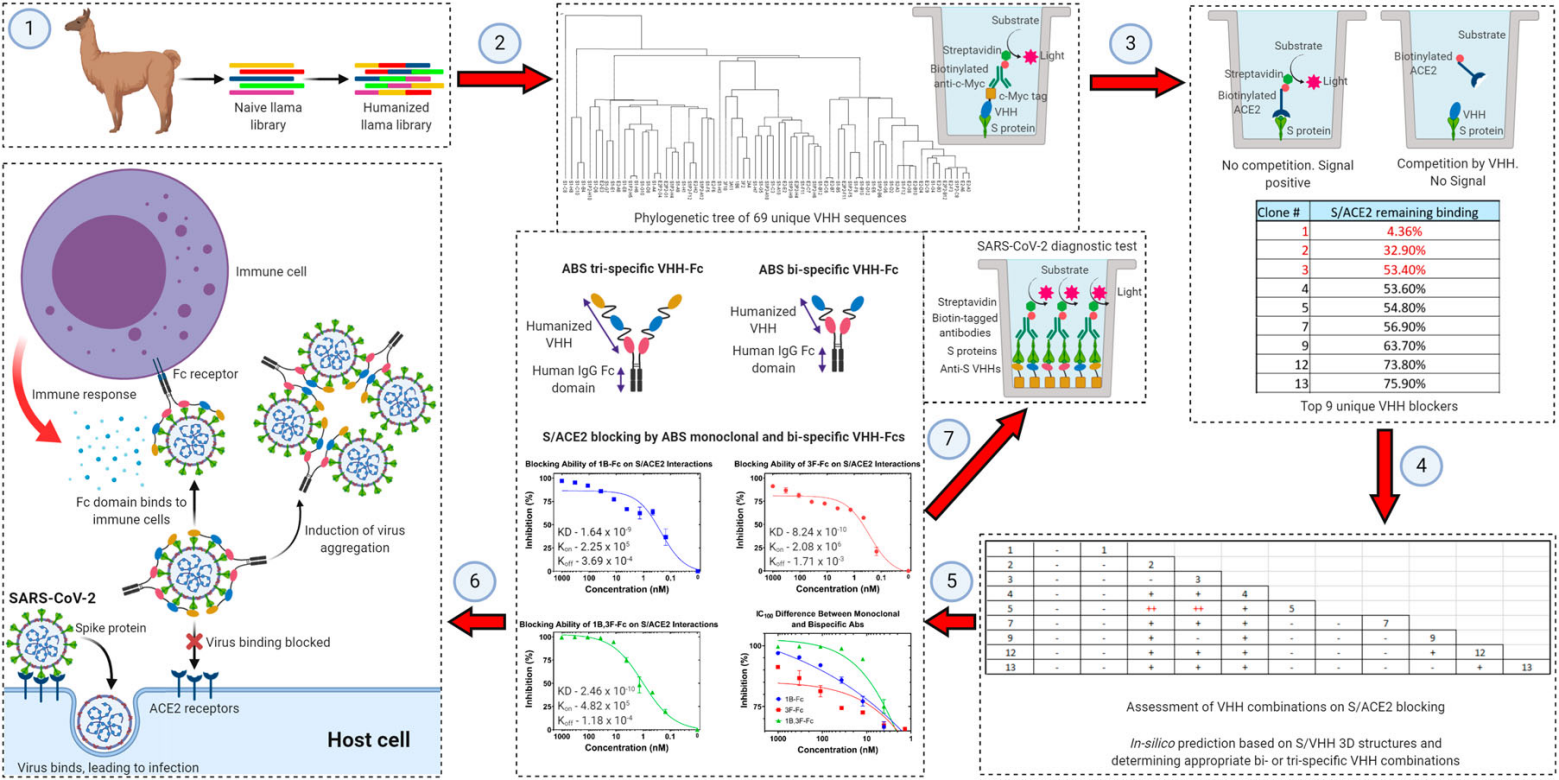
The workflow of anti-SARS-CoV-2 antibody discovery. (1) PBMC from 65 llamas were obtained, RNA was isolated, and cDNA was generated. Then, the VHH genes were amplified by two rounds of PCR and cloned to a phage display vector to construct the naïve VHH library. The synthetic VHH library was prepared by incorporation of shuffled VHH CDR1, 2 and 3, generated by overlapping PCR, to a modified human VH scaffold. (2) The VHH phage libraries were used for panning SARS-CoV-2 S1 fused to mouse Fc protein as the target antigen. Wells were coated with anti-mouse Fc to immobilize the antigen, and 3 rounds of phage panning were performed with reduced antigen concentration in each round. In ELISA assays, plates were coated with SARS-CoV-2 S1, the bound VHHs were detected by biotinylated anti-c-Myc antibodies and subsequent addition of streptavidin-HRP. The phylogenetic tree for 69 unique VHH binders is shown. (3) ELISA for ACE2 competition assay was performed by coating the plates with SARS-CoV-2 S1 as described previously and adding VHH in the presence of biotinylated ACE2. S1/ACE2 blocking function was determined by the reduction of HRP-induced chemiluminescence signal. The list of 9 unique S/ACE2 blockers is shown. (4) The ACE2 competition assay was repeated with a pairwise combination of the 9 S/ACE2 blockers, and the results are shown. (−): >=100%, (+): 80%−100%, and (++): <80% of the signal remaining compared to single VHH additions. Two VHH pairs have synergistic effects on blocking as shown in Red. (5) Structural organization of bi-specific and tri-specific llama VHH nanobody-Fc molecules that have been designed. The design process utilizes CAAD that optimizes features of VHH-Fcs. The concentration-dependent blocking of S/ACE2 binding by monoclonal (1B and 3F) and bi-specific (1B-3F) VHH-Fcs, and their IC100 differences are shown. The KD, K_on_ and K_off_ values for S protein binding by those antibodies are also shown. (6) Potential therapeutic mechanisms of ABS nanobody-Fcs. (7) Potential diagnostic utilization of humanized llama VHHs as single or combinatorial probes. (Created with BioRender.com).

By selecting and fusing two or three different humanized VHH sequences that target different but adjacent S protein RBD epitopes with high affinity and avidity into a single multi-specific antibody, while avoiding binding competition among the VHHs to S protein, we aim to improve overall S protein binding affinity and avidity and increase the S/ACE2 blocking function of our therapeutic antibodies. This design is aided by analysing different VHH combinations *in-silico* with modelled structures via our signature computer-aided antibody design (CAAD). We also use CAAD to further improve the VHH sequences so that the VHHs have low immunogenicity in humans, and high developability/manufacturability. The designed candidates are fused to human IgG Fc domains, and analysed *in-vitro* by binding and blocking assays to determine their S protein binding and S/ACE2 blocking capabilities, respectively. As expected, the bi-specific VHH-Fc antibody we have currently generated shows significantly better S protein binding and S/ACE2 blocking functions than each monoclonal VHH-Fc at therapeutically relevant concentrations, that is consistent in three independent attempts. It shows potent S/ACE2 blocking, with ∼100% blocking at 36.7 nM, and ∼95% blocking at 12.2 nM that will be important in successful elimination of the SARS-CoV-2 infection (Figure 1). We will test these multi-specific VHH-Fc antibodies and study their SARS-CoV-2 neutralizing capabilities with our collaborators. These molecules would potentially protect against SARS-CoV-2 by blocking S/ACE2 interaction and subsequent virus internalization, promoting virus aggregation, and inducing Fc-dependent antiviral functions, thereby possessing multiple mechanisms of action (Figure 1). In addition, these multi-specific antibodies would be easier to manufacture than polyclonal antibodies due to the production needs of only one molecule.

We will also assess the remaining 60 S protein high-affinity binders without S/ACE2 blocking function with our collaborators to probe whether they could be used for diagnostic applications to detect serum S protein and/or SARS-CoV-2 virions, either as single VHHs or in combination (Figure 1). Thus, we look forward to contributing in the fight against COVID-19 by developing antibody-based therapeutics and diagnostics.
